# Chest pain: when in doubt…

**DOI:** 10.1136/heartjnl-2019-316458

**Published:** 2020-04-14

**Authors:** Rong Bing, Andrew J Mitchell, David E Newby

**Affiliations:** 1 Edinburgh Heart Centre, Royal Infirmary of Edinburgh, Edinburgh, UK; 2 BHF Centre for Cardiovascular Science, University of Edinburgh, Edinburgh, UK

**Keywords:** acute myocardial infarction, cardiac catheterisation and angiography

## Clinical introduction

**Figure 1:**
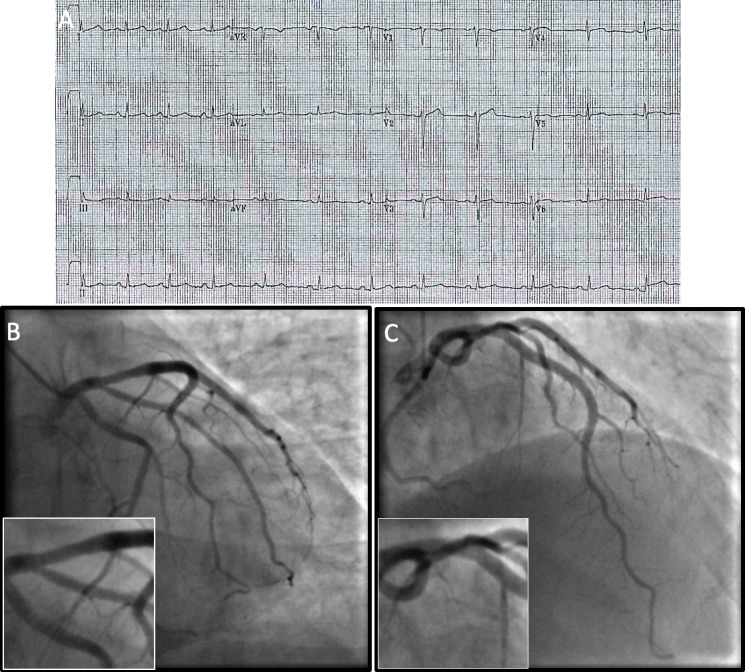
Index presentation. (A) ECG and (B) coronary angiography: right anterior oblique–caudal view. (C) Coronary angiography: anterior–posterior cranial view. Insets show the proximal left anterior descending artery.

A 38-year-old Caucasian man presented with central chest heaviness at rest. There was a history of mild abdominal discomfort but no infective prodrome. There was no antecedent exertional or nocturnal chest pain. The patient had a 26-pack-year history of tobacco smoking and no known comorbidities. Recent social history was unremarkable. Examination and vital signs were normal. An ECG taken with mild residual chest discomfort is shown in figure 1A. High-sensitivity troponin I was 74 ng/L, peaking at 1405 ng/L. Echocardiography demonstrated a structurally normal heart with normal biventricular function. Medical therapy for an acute coronary syndrome was commenced and invasive coronary angiography was performed (figure 1B, C).

## Question

What is the most likely diagnosis?

- Myocardial infarction with non-obstructed coronary arteries.
- Stress cardiomyopathy.
- Myocarditis.
- Microvascular dysfunction.
- Coronary artery spasm.

## Answer: A

### Discussion

In the era of high-sensitivity cardiac troponin assays, the aetiology of myocardial injury can be difficult to ascertain. In this case, myocardial infarction with non-obstructed coronary arteries^1^ due to plaque erosion was the probable cause of the index presentation, given the typical presentation and left anterior descending (LAD) atheroma. Stress cardiomyopathy was unlikely given the lack of a trigger, regional wall motion abnormality on echocardiography or ECG changes.^2^ Mild myocarditis was less likely in the absence of an infective prodrome and a clinical presentation most in keeping with acute ischaemia, although cardiac magnetic resonance (CMR) may be a useful test when there is clinical uncertainty. Microvascular dysfunction is a recognised cause of ischaemia^3^ but typically presents with a history of angina rather than a discrete episode of myocardial infarction. Similarly, coronary artery spasm usually presents with episodic chest pain of diurnal variation that responds rapidly to nitrates. If an ECG is taken during an acute episode, transient ST-segment deviation can be seen, not present in in this case.^4^ In situations of diagnostic ambiguity, the use of adjunctive tools such as intravascular imaging or CMR^5^ should be considered to clarify the underlying pathology and guide therapies.

**Figure 2:**
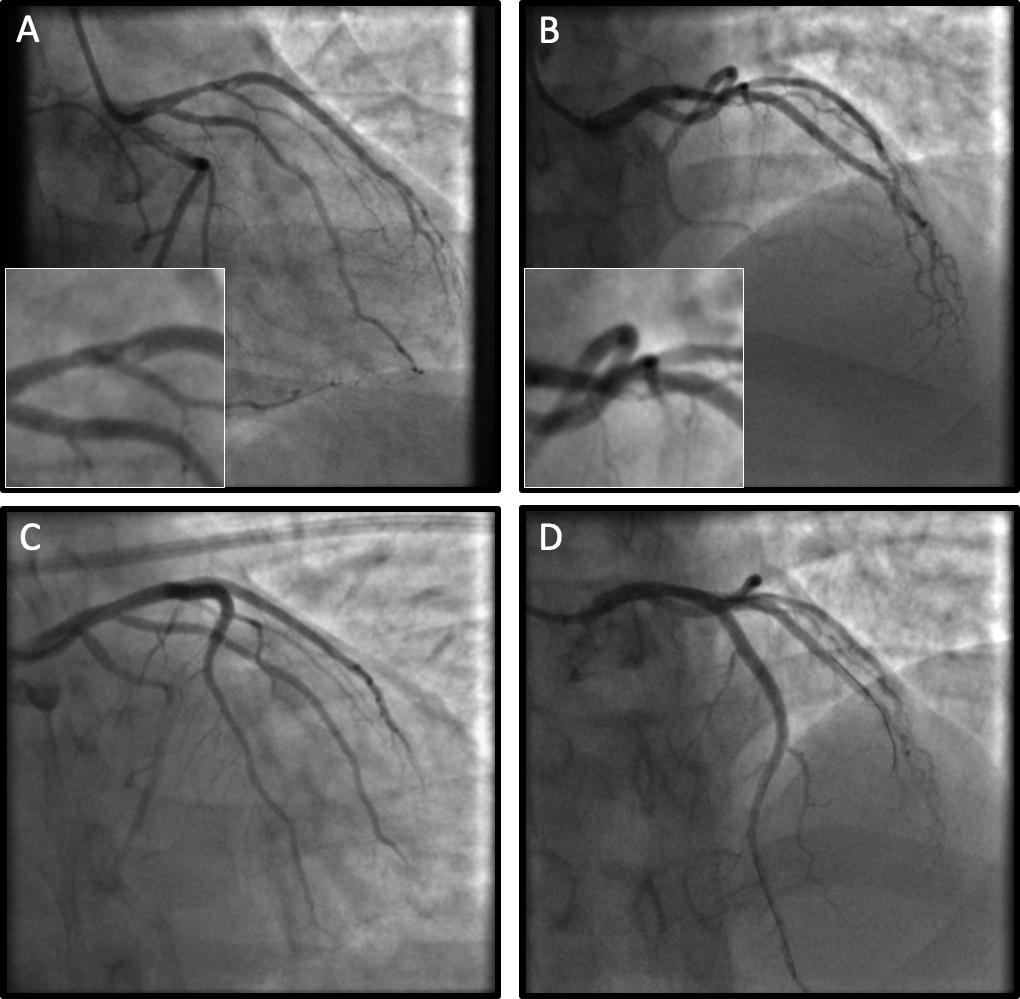
Angiography at re-presentation. (A–D) Right anterior oblique–caudal/cranial views showing mid-left anterior descending artery occlusion with progression of proximal left anterior descending artery disease and thrombus extending into the large second diagonal branch. (C, D) Post-percutaneous intervention views from the same projections demonstrating a recanalised left anterior descending artery.

However, myocarditis was felt to be the most likely diagnosis by the treating team. Preventative therapies were stopped and the patient was discharged. He continued to smoke. Six months later, he re-presented with severe chest pain and anterior ST elevation. Emergent coronary angiography demonstrated a thrombotic occlusion of the mid-LAD artery (figure 2A, B). Primary percutaneous intervention was undertaken (figure 2C, D). The patient recovered well.

## Patient and public involvement

This case was written without patient involvement. Patients were not invited to comment on the study design and were not consulted to develop patient relevant outcomes or interpret the results. Patients were not invited to contribute to the writing or editing of this document for readability or accuracy.
